# Fron-Mueller’s Case of Spontaneous Hæmorrhage from the Bulb of the Eye

**Published:** 1844-09-21

**Authors:** 


### FRON-MUELLER'S CASE OF SPONTANEOUS HÆMORRHAGE FROM THE BULB OF THE EYE.

F. M. was sent for to visit an operative who had been for some time labouring under the commencement of a staphyloma of the right eye, in consequence of a chronic ophthalmia of an arthritic nature. He found the patient seated with a basin before him, into which were falling drops of blood from the right eye. This hæmorrhage had supervened after violent pain of the eye, and had continued an hour. On examining the eye, there was discovered a red tumour, of the size of a pea, with a short peduncle, which protruded from the centre of the cornea, whence the blood proceeded. The tumour was excised near its base, by means of a pair of scissors. The hæmorrhage ceased after the operation. The tumour consisted of a membranous sac, apparently formed chiefly by the iris; it contained minute clots of blood. After the excision of the tumour, the bulb diminished in size; there remained four rugous marks corresponding to the recti muscles. The cornea lost its transparency.—*Ibid. from ibid.*

